# Decolonising and Diversifying Veterinary Education: Why It Matters and How to Begin

**DOI:** 10.1111/vop.70128

**Published:** 2026-01-05

**Authors:** Serena Maini

**Affiliations:** ^1^ Royal Veterinary College London UK

**Keywords:** colonialism, community, curriculum, ethnoveterinary, indigenous, knowledge

## Abstract

The decolonisation of veterinary education is an emerging topic in academic discourse. Introduced to the concept during a postgraduate certificate in veterinary education, I was surprised by how little literature or guidance existed on the subject. This article is not written from a place of authority, but from a desire to kindle an important conversation, one that examines how colonial legacies continue to shape veterinary curricula, research practices, and access to the profession itself.

Drawing on personal experience, blog posts, and scientific literature, I explore how knowledge hierarchies formed through colonialism continue to privilege Western science while sidelining indigenous and ethnoveterinary practices. From “helicopter research” to the underrepresentation of individuals of colour in the veterinary profession, this article traces how colonial dynamics remain embedded in the educational, research, and professional systems we often consider neutral or progressive. Rather than offering a prescriptive solution, this paper aims to spark curiosity, discussion, and action. It suggests that decolonisation begins with personal and institutional insight and reflection and emphasises that meaningful change requires humility, inclusion, and a willingness to confront discomfort. The goal is not to reject existing knowledge, but to enrich it by integrating perspectives that have long been ignored or undervalued. By opening up this discussion, I hope to contribute to a more honest, inclusive, and forward‐thinking vision of veterinary education, one that better serves a diverse global community of practitioners, researchers, and animal owners.

## 1 Viewpoint Article

In scientific literature, we are accustomed to seeking insight from established experts and leading researchers who have spent years refining their knowledge. But expertise is not the only driver of progress. This article is different. It is not the work of an expert; it is the start of a conversation we should be having.

Colonialism describes the systemic occupation, political control, and economic exploitation of indigenous lands and their people [1, 2]. With only five countries worldwide that escaped European colonialism or control (Japan, Korea, Thailand, Liberia, and arguably, Ethiopia [3]), its global effect cannot be ignored. However, colonialism was not just about land, power, and resources—it also shaped knowledge. It created hierarchies of expertise which validated some approaches and ignored others (“coloniality of knowledge”), causing a power imbalance weighted in Europe's favor [4, 5]. Education and academia as we practice them are rooted in colonial, Eurocentric institutional structures and, despite the dismantlement of colonial empires, the structures remain. Decolonising education means recognising colonial structures, dismantling the barriers they have created, and integrating diverse knowledge systems currently seen as “alternative” or “inferior.” Crucially, however, decolonisation is not about rejecting or erasing Western knowledge but about broadening our global understanding and asking: whose voices and perspectives are missing?

While conversations surrounding decolonisation have gained momentum in global health and international development, decolonisation of higher education is in its infancy, and that of veterinary education, barely conceived. The paucity of literature in this area serves to highlight the need for further work and reflects the main objective of this article: to widen awareness and understanding of decolonisation and to invite curiosity and discussion on how it may be achieved.

Echoes of colonialism play out in all levels of modern society, and animal health is no exception. Global animal health decision‐making often reflects colonial‐era hierarchies, with veterinary professionals from the Global North dictating policies and practices in regions historically subjected to colonial rule. This de‐localised approach perpetuates inequities and undermines the agency of local communities, leading to the extraction of research and labour for the benefit of privileged institutions [6]. Furthermore, it fails to recognise the value of indigenous knowledge and the cost of losing it.

Wildfires are a well‐known environmental example of the consequences of lost indigenous knowledge. For centuries, indigenous communities have practiced controlled burns to shape the landscape, maintain biodiverse ecosystems, and prevent catastrophic fires. However, colonial suppression of these methods, in addition to climate change, has contributed to an alarming increase in wildfires globally [7].

In the same way that ecosystems suffer from the exclusion of indigenous expertise, veterinary medicine loses out when certain ways of knowing are dismissed or devalued. Ethnoveterinary medicine encompasses traditional animal healthcare practices, including the use of medicinal plants and cultural rituals. These medications and practices, although sometimes lacking the scientific studies often considered ‘gold standard’ in the West, have been developed over centuries and are fine‐tuned to the local environment. Colonialism sidelined these methods in favour of Western veterinary medicine, often disregarding valuable, sustainable, and locally adapted practices. Ndou et al. [8] documented the use of 31 medicinal plant species by South African livestock owners for the management of animal health issues and indicated that indigenous remedies are at risk of disappearing due to Western influence. Participants were concerned about the ongoing erosion of ethnoveterinary knowledge and emphasised the need for its preservation. In India, local farmers and herders use plant extracts to treat infections and boost animal immunity, with results that reportedly rival Western pharmaceuticals [9]. But because these methods do not fit the Western scientific model, they are assumed to be of poor quality or inferior. Given the global pressures on veterinary healthcare, most notably antimicrobial resistance, can we afford to ignore diverse approaches? If these knowledge systems were embraced rather than dismissed, could they enrich veterinary medicine by providing solutions that are more holistic, locally accessible, and sustainable?

Another manifestation of Global North–Global South power dynamics in global health is the phenomenon of “helicopter research,” also known as “parachute” or “neocolonial” research. This refers to research conducted in the Global South by scholars from the Global North who collect data or samples from local communities, with minimal collaboration, credit, or benefit to the communities involved. In the veterinary and global health fields, this is evident in publications that barely incorporate the objectives of local researchers and exclude them from authorship, bypass local ethical standards, or fail to share results with the communities that contributed to them. Craddock [10] explores how One Health initiatives, while noble in their intention, can fall short in practice. Craddock highlights a case study on zoonotic African trypanosomiasis in Uganda that illustrates the disconnect between externally driven research and funding and the realities of local practices, limited resources, and government constraints. While One Health initiatives are a step in the right direction, Craddock encourages us to question: Whose health is being prioritised? And which economies are being secured?

Underrepresentation in research is compounded by structural barriers inherent in journal publication. Published research is more likely to be considered legitimate and of good quality if presented in English by predominantly white authors [11]. Global North researchers are often regarded as global experts, whereas their Global South counterparts are typically regionally confined, with their expertise validated only if it derives from the Global North. Cost‐prohibitive journal fees further limit the visibility of Global South research, effectively reserving open access publication as a privilege of the Global North [12].

This top‐down dynamic is not limited to research practices; it is also reflected in the underrepresentation of ethnic minorities across the veterinary profession. In the United States, Black Americans make up an estimated 13.7% of the population [13], yet comprise only 1.3% of the veterinary workforce [14]. This disparity is mirrored in specialist fields such as veterinary ophthalmology. Cross‐referencing the American Veterinary Medical Association's list of Black diplomates [15] with the American College of Veterinary Ophthalmologists' member data [16], Black representation within the ACVO remains similarly low at 1.3%.

While pan‐European statistics are more difficult to source due to inconsistencies in census categories, Wikipedia estimates that approximately 14% of Europe's population belongs to minority ethnic groups [17]. In contrast, an informal estimate suggests that just 2.1% of diplomates in the European College of Veterinary Ophthalmologists (ECVO) identify as non‐White [18, 19].

In the United Kingdom, non‐White individuals represent around 18.3% of the population in England and Wales (Census 2021), yet account for just 4.0% of Royal College of Veterinary Surgeons (RCVS) membership (RCVS 2024).

Lack of diversity in veterinary science reflects disparities that begin much earlier in the professional pipeline. Gamsu [20] examined ethnic diversity among university students in the United Kingdom and found veterinary science to be among the least diverse disciplines. In the 2014/15 academic year, 94.2% of UK veterinary students identified as white. Fewer than 55 students came from non‐White backgrounds and, of those, only 15 identified as Black Caribbean, Black African, Pakistani, Bangladeshi, Indian, or Chinese. The majority of students of colour were classified under “other” ethnic categories, not recorded in the national census.

Systemic imbalances in accessibility of higher education, research practices, and representation highlight just how deeply colonial legacies persist in the veterinary profession. Yet, despite the clear need for change, it is perhaps unsurprising that not everyone welcomes the idea of decolonisation, which can create tension and resistance [21, 22]. It is common for such topics to cause discomfort, and you may notice this in yourself as you read. If so, know that this response is a natural and understandable part of engaging meaningfully with the conversation. Most of us inherit a legacy tied to either formerly colonised communities or nations that once held colonial power. Our individual relationships with colonialism can be difficult to confront, especially for those who have benefited and continue to benefit from it. But discomfort is not something to be afraid of; it is a necessary step toward accepting historical truths and making meaningful change for the future.

My own relationship with colonialism, as a third‐generation Indian immigrant, stirs a complex mix of emotions—anxiety, fear, anger, sadness, disbelief, a yearning for justice, and hope. Throughout my childhood and early adulthood, I consciously embraced my Northern Irish identity—somewhat ironically, given Northern Ireland's own colonial history. I suspect this was my way of distancing myself from my colonised Indian heritage, which, even as a child, I sensed was perceived as “less than.” By increasing my proximity to whiteness, it felt as though I was increasing my proximity to legitimacy and acceptance. Heartbreakingly, this demonstrates that even as a child, I had imbibed the societal assumption that the Global South was inferior to the Global North. I spent much of my adult life searching for an identity that felt truly mine. Coming to terms with the historical and ongoing impacts of colonialism has been unsettling and uncomfortable, distressing even—but it has also helped me reconnect with my Indian heritage. Recognising the presence and impact of colonial legacies has given me a deeper sense of authenticity and purpose, and, rather than weighing me down, has become a source of energy, curiosity, and conviction.

Reflecting on my own experiences, I recognise not only how I have been harmed by colonialism but also how I, a British graduate, have perpetuated the harmful effects of colonialist paternalism. As a newly qualified vet, I spent a few weeks volunteering at a charity clinic in Peru in 2007. My peers and I (along with many other veterinary students in the United Kingdom) viewed this trip as a chance to “practice” surgical skills and gain confidence in neutering procedures before entering professional practice—a troubling perspective that reflects a problematic narrative upheld by some overseas veterinary volunteer charities. This narrative reinforces notions of superiority and indispensability among volunteers from the Global North, often sidelining local healthcare workers and potentially affecting patient care. I recall (with some shame) an afternoon in the Peruvian clinic when my colleagues and I were performing canine ovariohysterectomies while the harried local nurse ran from patient to patient “topping up” intravenous anesthetic. The realisation hit me, standing elbows deep in a stray dog's abdomen, looking at the dozen or so blood‐soaked swabs next to me, that I was not the right person to be doing this. Why were these unowned dogs suitable for us woefully inexperienced surgeons to “practice” on? They were not. They deserved better; in fact, they deserved the best. These patients deserved the most experienced surgeons, who were not just proficient in the procedure but also familiar with the local environment. The neutered dogs were discharged to the surrounding streets hours after their surgery, and the risk of postoperative complications should have been as close to nil as possible. This could not be achieved by new graduates with minimal surgical experience and scant understanding of the locale. Local healthcare providers also deserved better—respectful collaboration that valued their knowledge and experience, instead of being sidelined to create opportunities for Global North trainees.

Thankfully (for the patients, at least), some veterinary volunteer charities have introduced formal training programmes specifically designed for visiting students and new graduates. And while this is a step in the right direction, several ethical considerations may still be overlooked; for example, the potential for perpetuating paternalistic attitudes toward local healthcare workers and the possibility of lost training opportunities for homegrown vets [23]. Some third‐party “voluntourism” companies aim to make a profit from fees paid by visitors [24], which leaves me feeling particularly uneasy—financial exploitation being yet another tenet of colonialism.

This brings us to the question—how do we undo these harmful effects of colonization? How exactly can we achieve decolonisation in veterinary education, at both undergraduate and residency levels? The reality is, there is no prescriptive answer. The literature is sparse and, truthfully, we are all still exploring and learning. Drawing from a minimal source database, which includes podcast episodes and blog posts, a handful of published articles, and preliminary work at the Royal Veterinary College (personal communication: Dr. Christine Thuranira‐McKeever, PhD, Royal Veterinary College, November 25, 2024), here are some thoughts to consider:

### 1.1 Decolonise oneself

Questioning ourselves and critically reflecting on our answers is an important starting point for anyone wishing to begin the process of decolonisation [25].

### 1.2 Decolonize and diversify curricula

Nondiverse and nonrepresentative curricula have been linked to negative outcomes, including lower academic attainment, feelings of isolation, and disengagement among students from minority backgrounds [26, 27, 28, 29]. Diversifying curricula starts with recognising colonial structures, dismantling historic barriers, and re‐inserting excluded voices, for example, teaching about indigenous animal health practices, including Global South researchers, and discussing colonial histories in animal health policies. Source material, images, and case examples may be reconsidered to better represent an increasingly diverse student body.

### 1.3 Rethink Assessments

Ensure that the assessment format does not disproportionately compromise outcomes for minority candidates [30].

### 1.4 Be Impactful, Not Performative

There can be a disparity between the advertisement of diversity initiatives and impactful change. It is particularly important that there is a sense of institutional responsibility, and supportive leaders should be driving decolonisation efforts [11].

### 1.5 Amplify marginalised voices

Who gets to be seen as an expert? Enhance the representation of minority groups by amplifying the inclusion of their research contributions and increasing their presence as educators, researchers, and students [25, 31]. Consider what students could offer to curriculum development, and consider involving them in the process. As identified in the National Insititute of Health Research (2021), ‘any change concerning a population should coproduce the change with that population’ [11, 32].

### 1.6 Address institutional barriers

Admissions and hiring practices must actively create spaces where diverse students and faculty can thrive, for example, the promotion of interdisciplinary discussions on socioeconomic, political, and cultural topics.

### 1.7 Mindful volunteering

Ask your students to consider—are local vets involved and treated in a respectful way? Are patients benefiting from the initiative? Is the volunteer's presence empowering the community, or is it exclusively serving their own learning?

In conclusion, decolonising veterinary education is not “one and done,” but an ongoing, iterative process—one that requires sustained reflection, humility, and collaboration. It begins with examining how colonial legacies shape curricula, research practices, and professional structures. It means amplifying marginalised voices, valuing diverse knowledge systems, and fostering a profession that reflects the global community it serves. It is not about throwing out existing knowledge; rather, it is about expanding it by integrating voices and perspectives that have long been ignored and sidelined. In this way, I hope we can move toward a more sustainable, inclusive, and intellectually honest profession.

## Author Contributions

**Serena Maini:** conceptualization, investigation, writing – original draft, writing – review and editing.

## Disclosure

Artificial Intelligence (AI) statement: The author has not used AI to generate any part of the manuscript.

## Conflicts of Interest

The author has declares no conflicts of interest.

## Data Availability

Data sharing not applicable to this article as no datasets were generated or analysed during the current study.
